# Comparison of Sensory Observation and Somatosensory Stimulation in Mirror Neurons and the Sensorimotor Network: A Task-Based fMRI Study

**DOI:** 10.3389/fneur.2022.916990

**Published:** 2022-06-30

**Authors:** Zhiqing Zhou, Songmei Chen, Yuanli Li, Jingjun Zhao, Guanwu Li, Lei Chen, Yuwei Wu, Sicong Zhang, Xiaolong Shi, Xixi Chen, Shutian Xu, Meng Ren, Shixin Chang, Chunlei Shan

**Affiliations:** ^1^Center of Rehabilitation Medicine, Yueyang Hospital of Integrated Traditional Chinese and Western Medicine, Shanghai University of Traditional Chinese Medicine, Shanghai, China; ^2^School of Rehabilitation Science, Shanghai University of Traditional Chinese Medicine, Shanghai, China; ^3^Department of Rehabilitation Medicine, Shanghai No. 3 Rehabilitation Hospital, Shanghai, China; ^4^Engineering Research Center of Traditional Chinese Medicine Intelligent Rehabilitation, Ministry of Education, Shanghai, China; ^5^Department of Radiology, Yueyang Hospital of Integrated Traditional Chinese and Western Medicine, Shanghai University of Traditional Chinese Medicine, Shanghai, China

**Keywords:** sensory observation, somatosensory stimulation, mirror neurons, sensorimotor network, functional magnetic resonance imaging

## Abstract

**Objective:**

This study aimed to investigate brain plasticity by somatosensory stimulation (SS) and sensory observation (SO) based on mirror neuron and embodied cognition theory. Action observation therapy has been widely adopted for motor function improvement in post-stroke patients. However, it is uncertain whether the SO approach can also contribute to the recovery of sensorimotor function after stroke. In this study, we explored the therapeutic potential of SO for sensorimotor dysfunction and provided new evidence for neurorehabilitation.

**Methods:**

Twenty-six healthy right-handed adults (12 men and 14 women), aged 18–27 (mean, 22.12; SD, 2.12) years were included. All subjects were evaluated with task-based functional magnetic resonance imaging (fMRI) to discover the characteristics and differences in brain activation between SO and SS. We adopted a block design with two conditions during fMRI scanning: observing a sensory video of brushing (task condition A, defined as SO) and brushing subjects' right forearms while they watched a nonsense string (task condition B, defined as SS). One-sample *t*-tests were performed to identify brain regions and voxels activated for each task condition. A paired-sample *t*-test and conjunction analysis were performed to explore the differences and similarities between SO and SS.

**Results:**

The task-based fMRI showed that the bilateral postcentral gyrus, left precentral gyrus, bilateral middle temporal gyrus, right supramarginal gyrus, and left supplementary motor area were significantly activated during SO or SS. In addition to these brain regions, SO could also activate areas containing mirror neurons, like the left inferior parietal gyrus.

**Conclusion:**

SO could activate mirror neurons and sensorimotor network-related brain regions in healthy subjects like SS. Therefore, SO may be a promising novel therapeutic approach for sensorimotor dysfunction recovery in post-stroke patients.

## Introduction

Stroke is the third leading cause of disability in adults worldwide (1). Recovering sensorimotor function after stroke is a common and sometimes tricky problem in clinical rehabilitation (2). Conventional rehabilitation approaches include neural facilitation, motor relearning, sensory retraining, transcutaneous electrical nerve stimulation, and other techniques, that can promote recovery of sensorimotor function to some extent (3–5). However, stroke is often accompanied by damage to sensory pathways, limiting the effects of conventional rehabilitation modalities (6). Sensorimotor impairments remain a pervasive problem for post-stroke patients, with the recovery of upper-extremity function particularly recalcitrant to intervention (7). We need to explore and develop more effective rehabilitation strategies that supplement or replace traditional rehabilitation.

In recent years, rehabilitation methods based on mirror neuron theory such as mirror therapy, motor imagery, and action observation (AO) therapy have received more and more attention (8, 9). Mirror neurons are a distinct class of neurons that discharge both when performing a specific action and when observing the same or similar action (10). They are the essential neural substrate for action understanding, imitation, execution, and empathy (11, 12). The mirror neuron system (MNS) is the ensemble of cortical motor centers endowed with the mirror mechanism, with different functions depending on its anatomical location (10). In humans, the core MNS includes the inferior frontal gyrus (IFG), ventral premotor cortex, inferior parietal gyrus (IPG), and intraparietal sulcus (9, 13). In addition, the extended MNS involves additional brain areas, such as the insula, middle temporal gyrus (MTG), and somatosensory cortex, which connect to the core system (12). Studies confirm the existence of an action observation–action execution matching mechanism in specific regions of the MNS, which are located in the frontal and parietal lobes (14, 15). AO therapy facilitated the recovery of motor function in stroke patients by activating this specific MNS (16). When an action is understood, it will cause resonance in the observer's motor areas (17). Notably, AO therapy relies on visual feedback and emphasizes the kinesthetic experience of perceived motion to generate motor representations, facilitating action execution (18). As we know from embodied cognition theory, our bodily experience is primarily derived from the integration of sensory, perceptual, and motor signals and is mapped directly to the sensorimotor cortex (19). AO prompts visual perception to be mapped onto activated mirror neurons, forming a motor representation (9, 14). The bidirectional flow of perceptual and motor information then ultimately facilitates action execution (20). Thus, it can be argued that the mirror neuron theory of action understanding is one of the most influential examples of embodied cognition theory (21). AO therapy shows a “top-down” effect in neurorehabilitation by activating the MSN and causing a reorganization of motor representations at the central level (14). So far, AO therapy has been successfully applied to the rehabilitation of motor function for stroke patients, children with cerebral palsy, and individuals suffering from Parkinson's disease (22–24).

AO therapy activates the motor areas of the MSN to improve motor function by generating motor representations (18, 25). However, we cannot ignore the influence of sensory representations on motor function and the sensorimotor network when considering motor control and motor rehabilitation. Phenomena such as the rubber hand illusion (26, 27) and the mirror synaesthesia (28, 29), and phantom limb sensations (30) illustrate that sensory representations can also be embodied. Studies of phantom limb phenomena show that individuals continue to have awareness and experience sensations of bodily unity and continuity despite actual sensory and motor loss (31, 32). Similarly, the rubber hand illusion confirms that under multisensory stimulation, vision typically dominates somatic sensation and has the potential to perceive the virtual body as its own (33). These findings are essential evidence for embodied cognition theory, which posits that the neural systems that perceive those properties can represent semantic knowledge of perceptual properties (34). So, could sensory observation (SO) facilitate the formation of sensory representations of perceived sensory experiences and mapping to the sensory nervous system with the help of visual stimuli? Research has established that mirror neurons are both motor and sensory neurons (35). Therefore, based on the combination of mirror neurons and embodied cognition theory, it is reasonable to assume that SO may activate the sensory areas of the MSN and form sensory representations through understanding sensory information a priori. The activation of the sensory nervous system may further facilitate the integration of sensory and motor networks, which would be more conducive to the recovery of sensory-motor function. Nevertheless, our hypothesis remains to be tested.

The sensorimotor network is responsible for the control of somatic sensation and movement, characterized by strongly functional coupling to nearby areas (36). The motor component of the sensorimotor network included the primary motor cortex (M1) and caudal premotor, whereas the somatosensory component included the primary somatosensory cortex (S1) and most of the somatosensory area (Brodmann's 5L) (36). It has been demonstrated that peripheral nerve stimulation can modulate M1 excitability via the existing cortico-cortical connection between S1 and M1 or *via* direct projections from the thalamic nucleus (37, 38). Nasrallah et al. (39) have demonstrated that effective sensory input is critical to motor output, which regulates the integration of sensorimotor networks. Therefore, the improvement of sensory function through sensory input-based rehabilitation training may be of great significance to the recovery of motor function (40). Previous sensory rehabilitation investigation has focused on somatosensory stimulation (SS) through “bottom-up” peripheral sensory inputs (41). Nevertheless, the effect of SS may be compromised in cases where the sensory circuits are impaired (42). Mikkel et al. (43) showed that input of visual signals improved tactile discrimination and acuity. It is possible that visual stimulation promotes S1 activation and leads to increased excitability of M1, which induces brain plasticity (20). Therefore, we wondered whether SO forming a subjective experience could produce effects similar to sensory stimulation. Could these “top-down” sensory representations activate sensory or motor areas in the sensorimotor network and thus facilitate sensory-motor integration? To date, SO and sensorimotor rehabilitation mechanisms have not been fully elaborated.

With the development of neuroimaging techniques, task-based functional magnetic resonance imaging (fMRI) has been employed to investigate neuroanatomical and functional changes in sensation and movement (44). It provides a good foundation for our research on the activation of mirror neurons and the sensorimotor network during SO (45). This study designed SO and SS as tasks to reveal the possible neural mechanisms underlying the sensorimotor function changes and their relevance with mirror neurons in healthy adults by a task-based fMRI. We hypothesized that SO therapy has the potential to treat sensorimotor dysfunction based on mirror neuron and embodied cognition theory. We also expect SO to activate the specific mirror neurons and sensorimotor network-related brain regions to enhance sensorimotor integration, which is a crucial hub for sensorimotor rehabilitation. It will provide a basis for further research on SO in post-stroke patients.

## Materials and Methods

### Participants

We recruited 30 undergraduate or graduate Chinese students as healthy participants (15 men and 15 women; age range, 18–28 years; mean ± SD, 22.57 ± 2.49 years) for this study through poster advertisements and word-of-mouth advertising. All participants were right-handed (Edinburgh handedness inventory) (46), had normal or corrected-to-normal vision, had no history of neurological or orthopedic diseases or drug or alcohol abuse and were not pregnant or lactating. This study was approved by the ethics committee at Yueyang Hospital of Integrated Traditional Chinese and Western Medicine, Shanghai University of Chinese Traditional Medicine, China (Ethics No. 2020-178). A total of 30 participants provided written informed consent, and this study was performed following the Declaration of Helsinki guidelines.

### Experimental Design

The study design was based on a block design and involved two conditions, as follows: (i) a video of a brush brushing someone's right forearm (task condition A) and (ii) a picture with a white circle on a black background (task condition B). There was a 30-s rest period between the two task conditions, during which a picture with a white fixation cross on a black background (rest period) was shown. When the brushing video appeared on the screen, participants were instructed to observe the brushing movements. When the picture with a white circle or fixation cross on a black background was visible, they were instructed to remain still, to think of nothing, and to focus on the screen. The difference between the task conditions was that, when the picture with a white circle appeared, personnel used a brush to brush the participants' right forearm. The brushing during task condition B was performed by the same professionally trained physiotherapist. Each task condition block was repeated twice in the ABBA sequence, with a rest period block in between each task condition block (9 blocks = 2 task condition A blocks + 2 task condition B blocks + 5 rest period blocks). Each block lasted for 30 s. Therefore, the total duration of the task was 4 min and 30 s.

**Figure 1:**
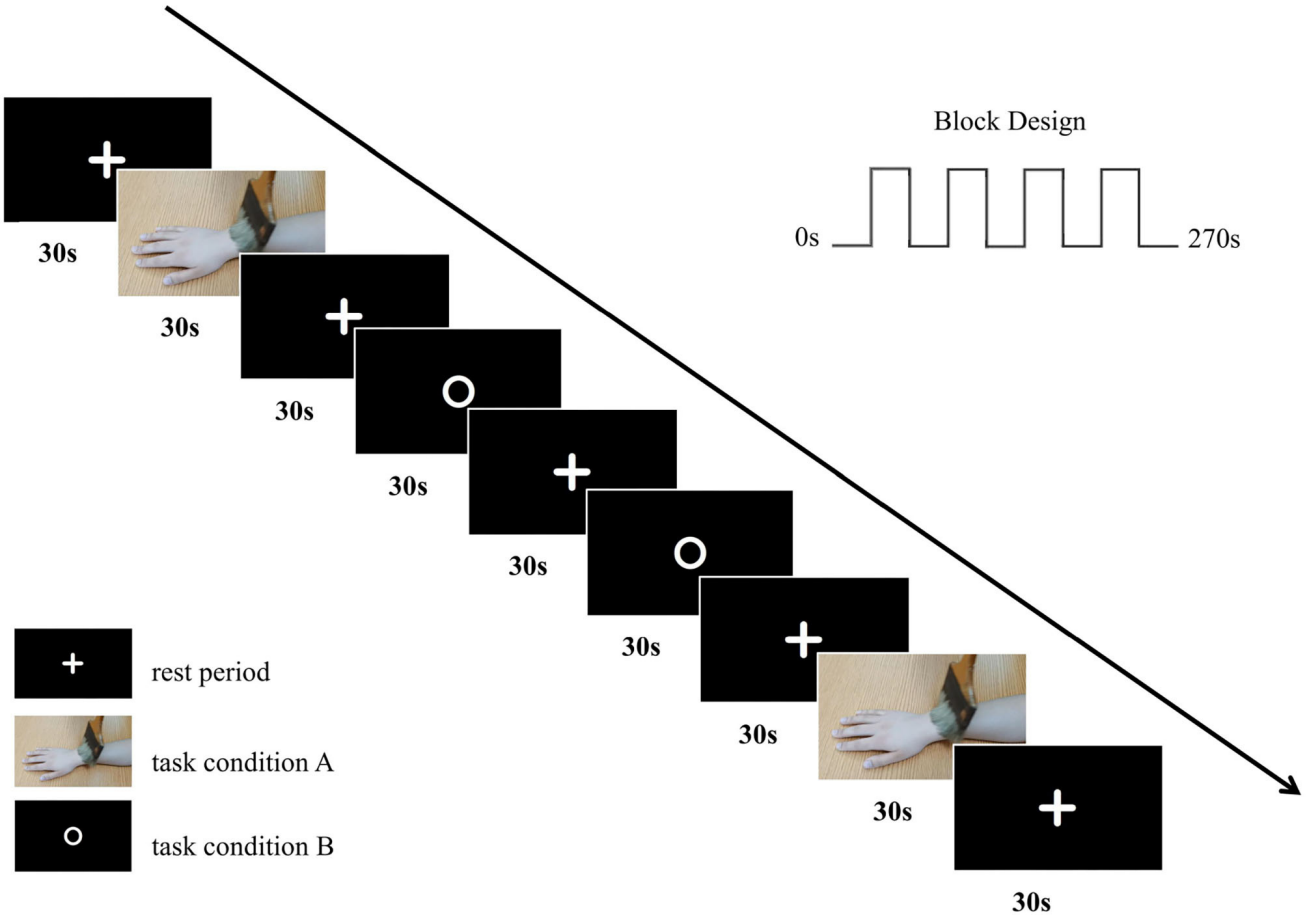
The basic fMRI experimental scheme. We adopted a block design with two conditions in our fMRI experiment with an ABBA task sequence.

Before fMRI scanning, participants were familiarized with the fMRI task. After a task-based scan, participants were instructed to close their eyes and keep their heads still, and a T1-weighted structural scan was collected immediately. The basic fMRI experimental scheme is illustrated in Figure 1.

### MRI Data Acquisition

Each participant underwent a series of scans using a 3-Tesla MRI scanner (SIEMENS VERIO, Erlangen, Germany) with an 8-channel head coil at Yueyang Hospital of Integrated Traditional Chinese and Western Medicine, Shanghai University of Chinese Traditional Medicine, China. They were positioned supine in the MRI scanner with a foam pad placed around their head to reduce movement and provided with earplugs to minimize the MRI noise. Stimulus presentation was controlled by Presentation software (SA-9939, http://www.sinorad.com/pro-show-103.html).

The MRI scanning session included a 4.5-min eyes-open task-based scan and a 6-min eyes-closed T1-weighted structural scan. Task-based fMRI images were obtained using the following sequence: repetition time (TR) = 2,500 ms; echo time (TE) = 30 ms; field of view (FOV) = 192 mm × 192 mm; flip angle (FA) = 90°; acquisition matrix size = 64 × 64; voxel size = 3 mm × 3 mm × 3 mm; number of slices = 39; slice thickness = 3 mm, with no gap. Three-dimensional magnetization prepared rapidly acquired gradient echo (MPRAGE) T1-weighted structural images were obtained using the following sequence: TR = 1,900 ms; TE = 2.93 ms; FOV = 256 mm × 256 mm; FA = 9°; acquisition matrix size = 256 × 256; voxel size = 1 mm × 1 mm × 1 mm; number of slices = 160; slice thickness = 1 mm, with no gap.

### fMRI Data Preprocessing and Analyses

The fMRI data were preprocessed and analyzed using the SPM12 software program (Statistical Parametric Mapping; https://www.fil.ion.ucl.ac.uk/spm/). First, original DICOM data were converted to NIFTI format, and their quality was checked. Two participants were eliminated in the follow-up phase due to insufficient data quality. Then, slice timing correction and motion correction (realignment) were performed using a middle slice as a reference (slice 39), and we excluded participants with excessive head motion (translation > 2 mm; rotation > 2°). Two participants were discarded due to excessive head motion. Next, we normalized the realigned images using the Montreal Neurological Institute (MNI)—T1 template (resampling voxel size, 3 mm × 3 mm × 3 mm). Finally, an 8-mm full width at half-maximum (FWHM) Gaussian kernel was applied to smooth the data.

After preprocessing, 26 participants were included in the first-level and second-level general linear model (GLM) analyses. For the first-level GLM analysis, we compared task condition A vs. the rest period and task condition B vs. the rest period. We defined task condition A vs. the rest period as SO and task condition B vs. the rest period as SS. Additionally, six head motion parameters obtained from the realignment step were used as covariates in the first-level analysis. For the second-level GLM analysis, we performed a one-sample *t*-test (two-sided) to explore the brain activation pattern under two conditions, with age and gender as covariates of no interest. Comparisons were conducted using paired-sample *t*-tests and conjunction analysis to determine differential and similar activations between SO and SS.

Results were visualized with xjView software (https://www.alivelearn.net/xjview/) and MRIcron software (https://www.nitrc.org/projects/mricron/). Only brain regions that survived a false discovery rate (FDR) corrected threshold of *p* < 0.001 at the voxel-level and a cluster size >10 voxels were reported.

## Results

### Whole-Brain Activation During SO

**Figure 2:**
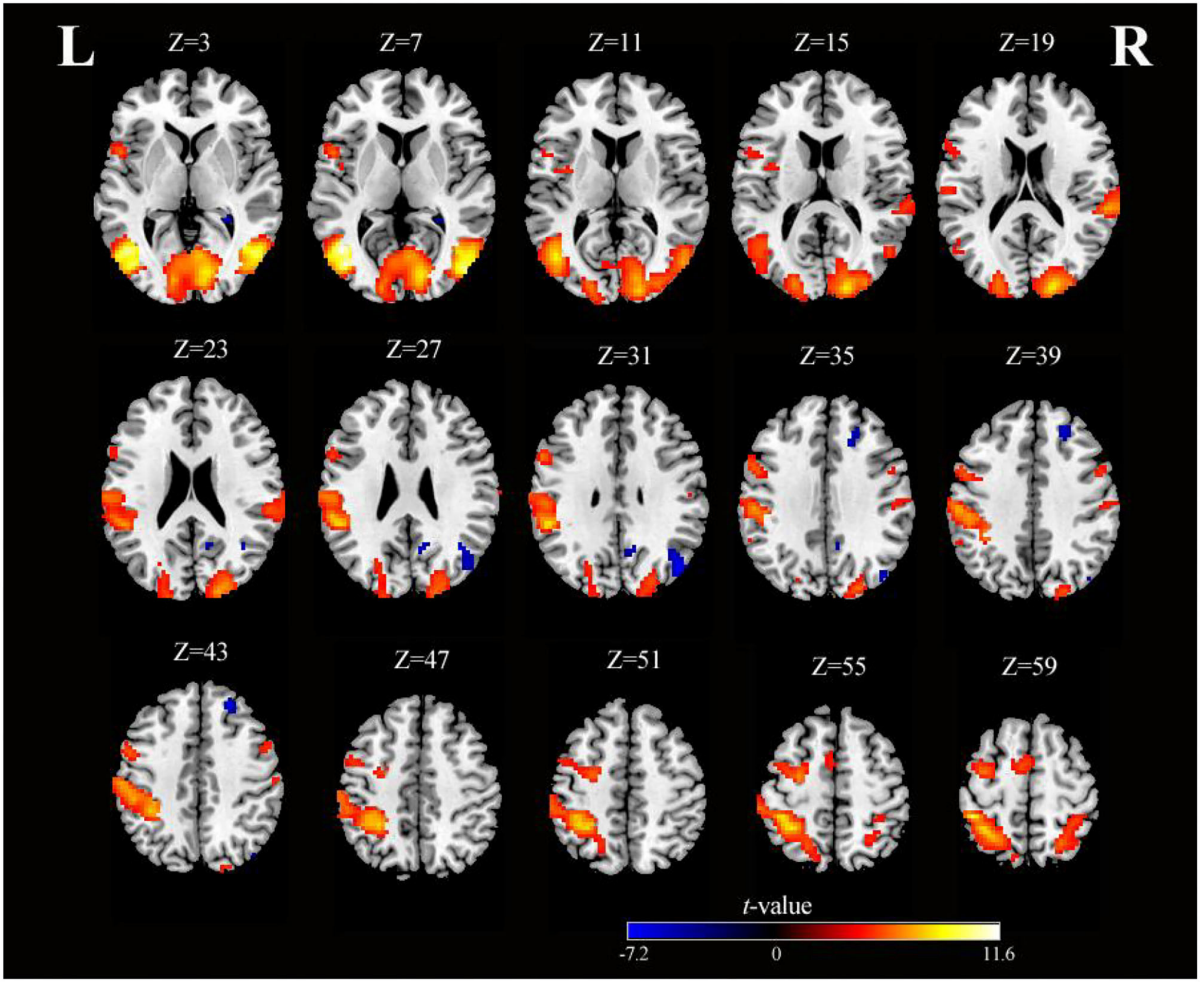
Axial slices with corresponding *Z*-coordinates (MNI) from the *t*-value map of SO (voxel-level, FDR-corrected *p* < 0.001, cluster size > 10 voxels). *MNI, Montreal Neurological Institute; SO, sensory observation; FDR, false discovery rate; L, left; R, right*.

As shown in Table 1 and Figure 2, SO activated the bilateral precentral gyrus (PreCG), left supplementary motor area (SMA), right postcentral gyrus (PoCG), left IPG, right superior parietal gyrus (SPG), right superior temporal gyrus (STG), left fusiform, right lingual gyrus, and left rolandic operculum (voxel-level, FDR-corrected *p* < 0.001, cluster size > 10 voxels).

**Table 1:** Brain regions with significant differences based on a one-sample *t*-test (two-sided) of SO (voxel-level, FDR-corrected *p* < 0.001, cluster size > 10 voxels).

**Brain regions (AAL)**	**Cluster size**	**Peak MNI coordinates (mm)**			**Peak *t*-value**
		**x**	**y**	**z**	
**Positive**
Lingual_R	2,885	−42	−75	6	11.6111
Parietal_Inf_L	1,030	−42	−39	60	9.5659
Precentral_L	306	−27	−9	54	6.6405
Parietal_Sup_R	136	24	−60	60	6.2609
Temporal_Sup_R	120	63	−33	21	7.6118
Supp_Motor_Area_L	108	−6	−3	66	6.1263
Fusiform_L	47	−42	−45	−18	6.6534
Postcentral_R	24	57	−21	39	4.8478
Precentral_R	22	51	6	42	5.1614
Rolandic_Oper_L	21	−36	−3	12	4.9797
**Negative**
Angular_R	61	45	−75	33	−7.2183
Frontal_Sup_R	34	24	39	42	−6.2509
Hippocampus_R	21	30	−36	−3	−6.1587
Precuneus_R	18	12	−60	27	−5.2053

*SO, sensory observation; FDR, false discovery rate; AAL, automated anatomical labeling; MNI, Montreal Neurological Institute; L, left; R, right*.

### Whole-Brain Activation During SS

**Figure 3:**
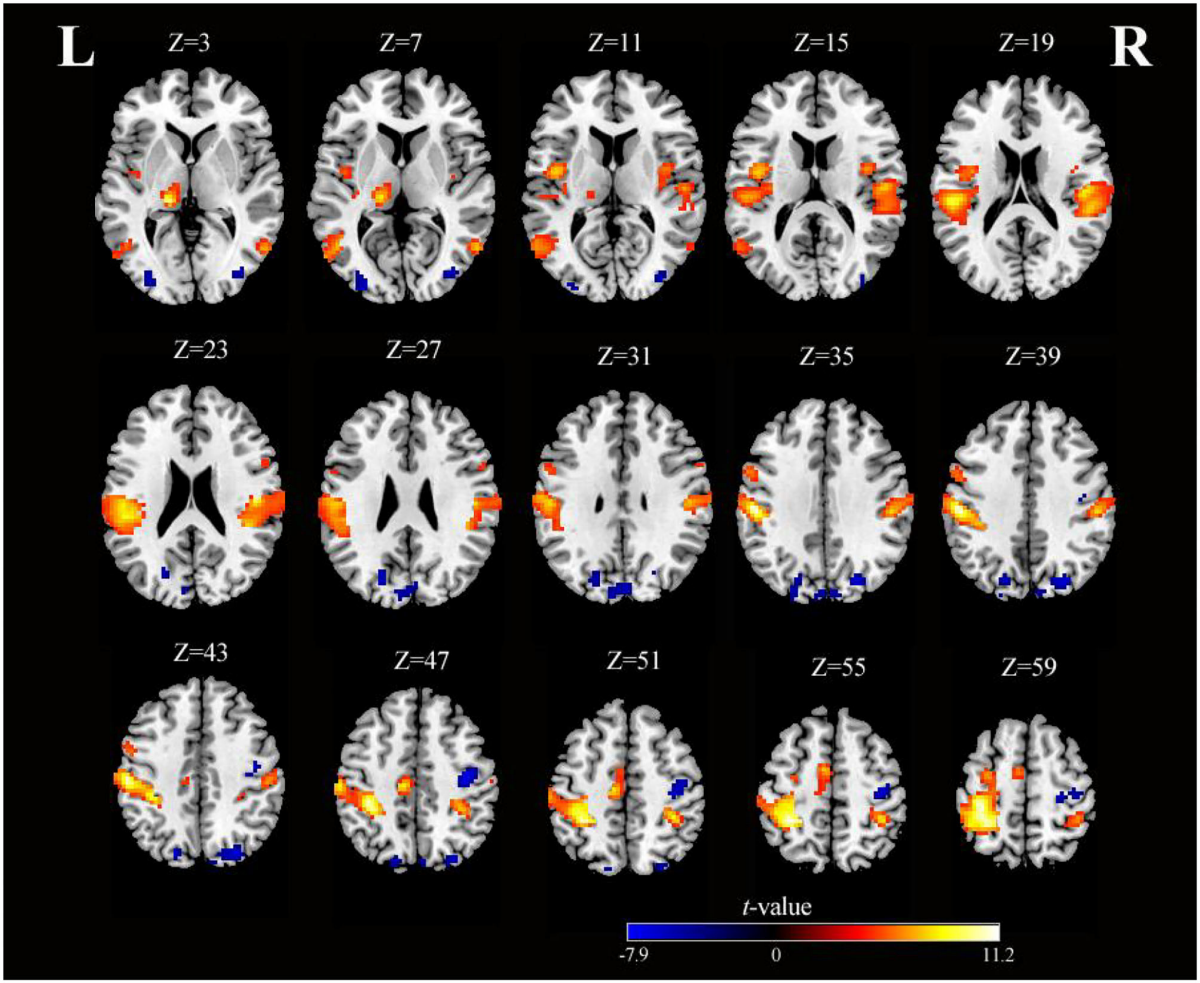
Axial slices with corresponding Z-coordinates (MNI) from the *t*-value map of SS (voxel-level, FDR-corrected *p* < 0.001, cluster size > 10 voxels). *MNI, Montreal Neurological Institute; SS, somatosensory stimulation; FDR, false discovery rate; L, left; R, right*.

As shown in Table 2 and Figure 3, SS activated the right supramarginal gyrus (SMG), bilateral MTG, left PreCG, left thalamus, right insula, right IFG (opercular part), and right superior cerebellum (voxel-level, FDR-corrected *p* < 0.001, cluster size > 10 voxels). Moreover, there were larger clusters of activation in the bilateral PoCG.

**Table 2:** Brain regions with significant differences based on a one-sample *t*-test (two-sided) of SS (voxel-level, FDR-corrected *p* < 0.001, cluster size >10 voxels).

**Brain regions (AAL)**	**Cluster size**	**Peak MNI coordinates (mm)**			**Peak *t*-value**
		**x**	**y**	**z**	
**Positive**
Postcentral_L	1,611	−33	−39	57	11.1946
SupraMarginal_R	443	48	−18	24	9.0303
Postcentral_R	131	36	−39	51	8.6381
Cerebelum_6_R	126	30	−45	−24	8.7086
Temporal_Mid_L	113	−48	−60	6	7.1451
Thalamus_L	75	−15	−27	3	9.9551
Precentral_L	52	−57	6	36	6.3694
Temporal_Mid_R	47	57	−63	6	7.2924
Insula_R	44	39	−3	15	6.5018
Frontal_Inf_Oper_R	13	57	9	27	5.0783
**Negative**
Precentral_R	115	36	−15	45	−7.8682
Cuneus_L	84	3	−87	33	−6.1839
Occipital_Mid_L	77	−39	−84	−3	−5.9007
Occipital_Sup_R	75	27	−75	36	−6.8805
Occipital_Sup_L	72	−21	−72	24	−6.0855
Occipital_Mid_R	44	36	−81	6	−5.7753
Cerebelum_4_5_L	23	−15	−51	−21	−5.6422

*SS, somatosensory stimulation; FDR, false discovery rate; AAL, automated anatomical labeling; MNI, Montreal Neurological Institute; L, left; R, right*.

### The Differences in Whole-Brain Activation During SO vs. SS

**Figure 4:**
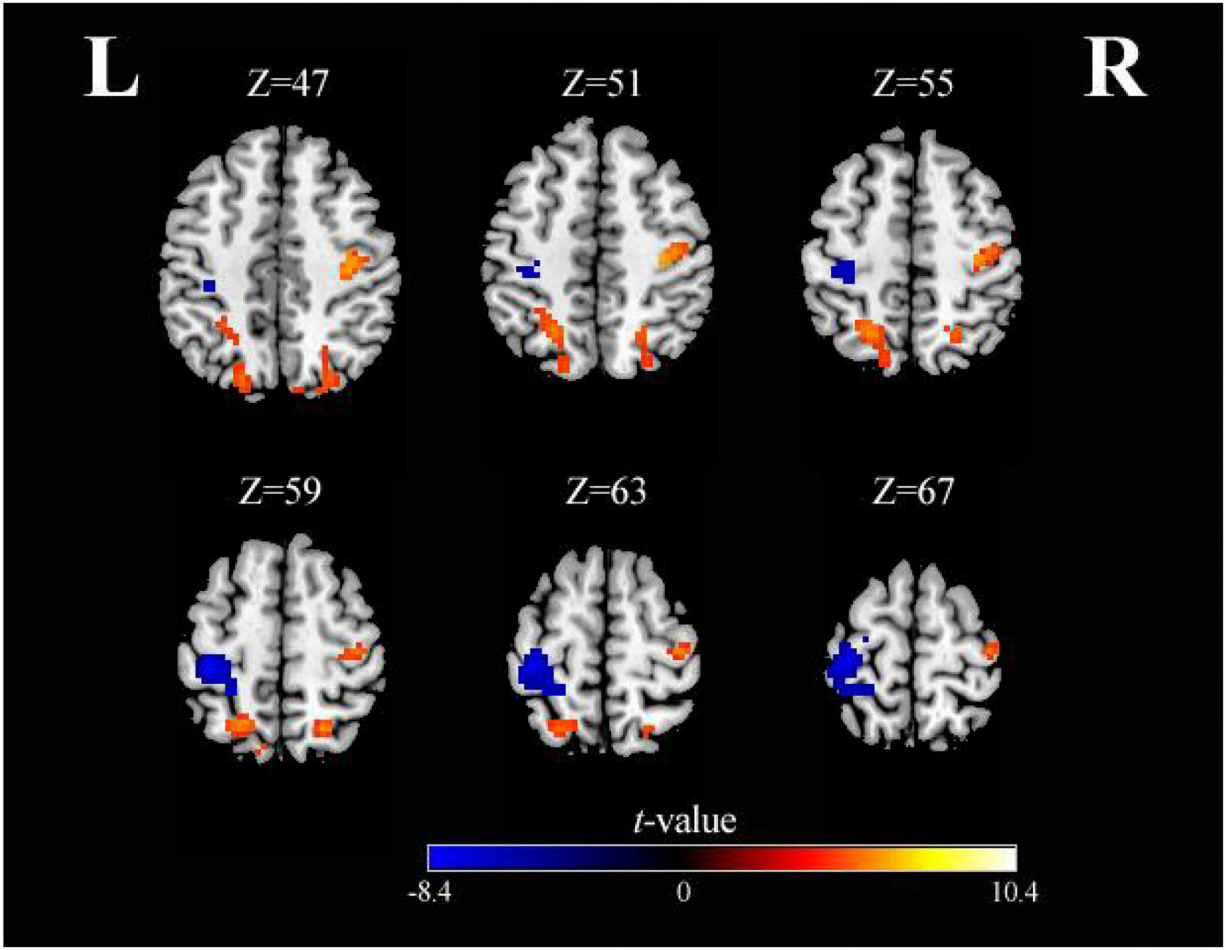
Axial slices with corresponding Z-coordinates (MNI) from the *t*-value map for differential activation of SO and SS (voxel-level, FDR-corrected *p* < 0.001, cluster size > 10 voxels). *MNI, Montreal Neurological Institute; SO, sensory observation; SS, somatosensory stimulation; FDR, false discovery rate; L, left; R, right*.

As shown in Table 3 and Figure 4, activation differences showed that SO activated PreCG more strongly than SS (voxel-level, FDR-corrected *p* < 0.001, cluster size >10 voxels). Conversely, brain regions of PoCG showed greater activation in SS than in SO (voxel-level, FDR-corrected *p* < 0.001, cluster size >10 voxels).

**Table 3:** Brain regions with significant differences based on a paired-sample *t*-test of SO and SS (voxel-level, FDR-corrected *p* < 0.001, cluster size > 10 voxels).

**Brain regions (AAL)**	**Cluster size**	**Peak MNI coordinates (mm)**			**Peak *t*-value**
		**x**	**y**	**z**	
**Positive**
Occipital_Mid_L	2,767	42	−81	6	10.3526
Precentral_R	88	36	−15	45	6.8359
**Negative**
Postcentral_L	231	−33	−27	72	−8.4383
Rolandic_Oper_L	34	−48	−27	21	−6.6247
Rolandic_Oper_R	16	45	−21	18	−6.3004

*SO, sensory observation; SS, somatosensory stimulation; FDR, false discovery rate; AAL, automated anatomical labeling; MNI, Montreal Neurological Institute; L, left; R, right*.

### The Similarities in Whole-Brain Activation During SO vs. SS

**Figure 5:**
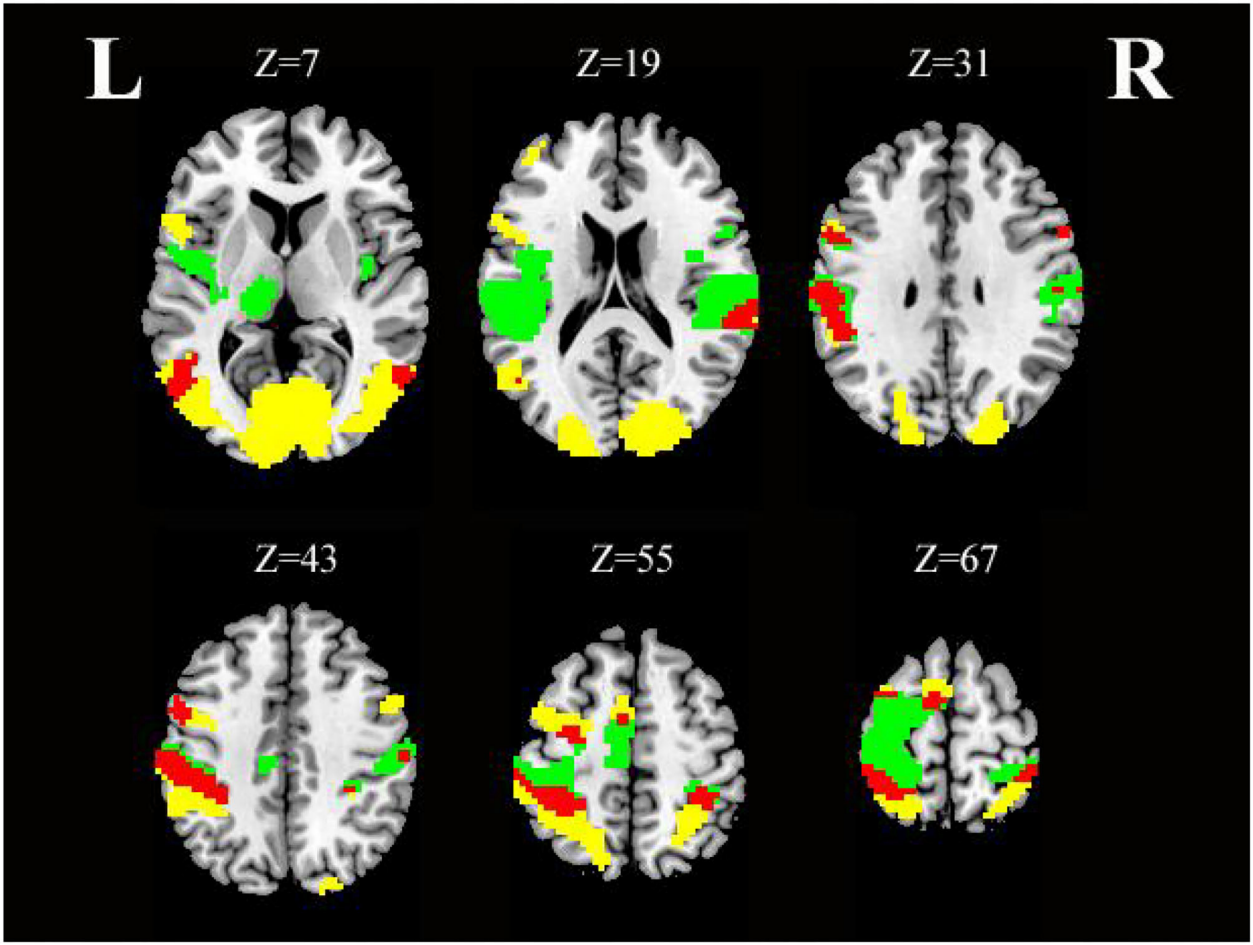
Axial slices with corresponding *Z*-coordinates (MNI) from the *t*-value map for similar activation of SO and SS; yellow activation shows areas recruited by SO, green activation shows areas recruited by SS, and red areas represent activation overlap between SO and SS, respectively (voxel-level, FDR-corrected *p* < 0.001, cluster size > 10 voxels). *MNI, Montreal Neurological Institute; SO, sensory observation; SS, somatosensory stimulation; FDR, false discovery rate; L, left; R, right*.

As shown in Table 4 and Figure 5, conjunction analysis revealed that both tasks activated the bilateral PoCG, left PreCG, bilateral MTG, right SMG, and left SMA (voxel-level, FDR-corrected *p* < 0.001, cluster size >10 voxels).

**Table 4:** Brain regions with significant differences based on a conjunction analysis of SO and SS (voxel-level, FDR-corrected *p* < 0.001, cluster size >10 voxels).

**Brain regions (AAL)**	**Cluster size**	**Peak MNI coordinates (mm)**			**Peak *t*-value**
		**x**	**y**	**z**	
Postcentral_L	351	−33	−45	54	8.4509
Temporal_Mid_L	61	−48	−63	9	6.4086
Precentral_L	37	−57	9	33	5.6978
Postcentral_R	34	36	−42	57	5.8526
Precentral_L	24	−27	−9	57	6.8952
SupraMarginal_R	21	63	−30	18	5.6565
Supp_Motor_Area_L	19	−6	−3	57	5.7551
Temporal_Mid_R	14	57	−60	6	5.5967

*SO, sensory observation; SS, somatosensory stimulation; FDR, false discovery rate; AAL, automated anatomical labeling; MNI, Montreal Neurological Institute; L, left; R, right*.

## Discussion

Neurorehabilitation therapies based on mirror neuron and embodied cognition theory play a vital role in motor dysfunction after stroke and are widely used in clinical rehabilitation nowadays (11, 47, 48). However, insufficient attention has been paid to sensory function rehabilitation. We believe that motor and sensory functions are inseparable parts of neurorehabilitation. Therefore, this study compared the effects of SO and SS on mirror neurons and the sensorimotor network-related brain regions in healthy subjects, which would provide more possibilities for the recovery of sensorimotor dysfunction after stroke.

A previous study has reported that observing someone's fingers being touched causes S1 to be activated (49). Another study has reported that SO could activate the secondary somatosensory cortex (S2) but not the S1 (50). In our SO task, we confirmed activation in PoCG, which is consistent with our hypothesis but inconsistent with the findings reported in a study by Chan et al. of 40 healthy participants (51). However, it has been controversial whether the somatosensory cortices can be activated by SO. Chan et al. thought that previous studies may have misattributed the activation of the somatosensory cortices because of the proximity (51), which may be one of the reasons for the controversy. Furthermore, we identified that, in some studies, the somatosensory cortex could be activated during SO, and their results were assessed at an uncorrected threshold with a small sample size (i.e., the number of subjects included in the analysis ≤ 15) (49, 52). A neuroimaging study has demonstrated the importance of multiple-comparison correction, and false-positive results can be controlled as much as possible with ≥25 subjects (53). In this study, 30 healthy participants were recruited, and 26 participants were included in the final analysis. Moreover, all our results were FDR-corrected. Only a significant FDR-corrected threshold of *p* < 0.001 at the voxel-level and a cluster size >10 voxels were reported. This may imply that our results have more strength. It should also be noted that these studies observed different parts of the body being touched, such as the legs, hands, fingers, and face (49, 50, 52, 54). Although SS of different body parts can lead to different sensations, it is not yet conclusive whether SO of different body parts can produce different sensory representations.

Maintaining the integrity of the sensorimotor network is the critical basis for maintaining sensorimotor function. We found that the bilateral PoCG, left PreCG, and left SMA, which are parts of the sensorimotor network, were activated in a conjunction analysis with SS and SO conditions. The activated brain regions are mainly concentrated in the left hemisphere because SS or SO of visual stimulation was performed on the subjects' right forearm. Katharina et al. revealed that a large part of S2 receives both ipsilateral and contralateral stimulation (55). Another study suggested that S2 may have hemispheric dominance, with different functional divisions between the left and right S2, regardless of the subjects' handedness (56). However, in this study, SS of the right forearm of the right-handed subjects could activate the bilateral PoCG and recruit more neurons in the left PoCG. Whether this is because of hemispheric dominance for sensory remains to be determined in future studies. Notably, there was a small cluster of activation in PoCG during SO, but there were large clusters in PreCG. The results of differential activation also showed that SO activated clusters in the PreCG more than the PoCG, while SS activated areas in the PoCG more. These findings are not difficult to understand. Indeed, SO is part of AO, which aims to observe the action of brushing but could make up for the lack of sensory representation. Moreover, SO therapy is more convenient to carry out and less demanding on equipment. It can easily be implemented in the clinic or even at home. The SO video can be played on nearly any electronic device, such as a mobile phone, tablet, computer, and even a television. Thus, we suggest that SO therapy, as a special form of AO therapy, has therapeutic potential for use in the treatment of sensorimotor dysfunction.

As expected, the IPG, PreCG, SPG, SMA, and PoCG can be activated in SO, suggesting that SO may be able to modulate the sensorimotor network by activating the MNS. This also provides a theoretical basis for SO therapy in neurorehabilitation. IPG and SPG, as important components of the posterior parietal cortex, are involved in information integration, such as the selection, preparation, and execution of movements (57, 58). Rozzi et al. determined that mirror neurons are mainly distributed in the inferior parietal lobule, which is very important for action organization and action understanding (59). We observed significantly strong activations in IPG, which is consistent with the findings of Rozzi et al. (59). From this, we hypothesized that the IPG may be a central hub of the mirror neuron circuits, and the entire MNS may be tightly connected through the IPG. However, little is known at this time about mirror neuron circuits and the neural mechanisms underlying MNS activation by SO. Substantial further research is required to determine whether post-stroke patients have the same activation patterns in the brain as healthy people.

Meanwhile, SS showed increased activation of the thalamus and insula. In particular, the thalamus is thought to be the final gateway for sensory input to the cerebral cortex and plays a crucial role in the sensorimotor circuits (60, 61). A recent study reported that increased gray matter volume in the thalamus is positively correlated with the recovery of motor function after stroke (62). Persistent functional reorganization within the neural networks may underlie the motor recovery process after stroke, and activation of cortical circuits or thalamic circuits may help to induce neural network plasticity (63). In addition to the thalamus, the insula was also activated by SS. In the macaque monkey literature, the insula is considered an integration center of motor, sensory, emotional, and social information (64). In humans, the insula has extensive connections with the frontal, parietal, temporal, and limbic regions, which are thought of as the key components of neural circuitry (65). It is regrettable that no significant activation was observed, which may be related to the visual input rather than somatosensory input during SO. Future studies should explore the effects of SO on brain structure and function in patients in order to better understand the neural mechanism for sensorimotor dysfunction recovery in post-stroke patients. It is also worth determining in the future whether the combined treatment of SO and SS is more effective than each therapy alone.

As far as we know, this is the first study to compare the effects of forearm SO and SS on mirror neurons and sensorimotor networks in healthy individuals. This study provides preliminary results in SO and hopes to enrich and refine the theory of mirror neurons and embodied cognition. On the other hand, this study also emphasizes the importance of the sensory process in motor function recovery. We will continue to explore the effects of multisensory integration on brain activation in sensorimotor-related regions in future studies so as to develop more precise and individualized rehabilitation programs for patients after stroke.

## Limitations

This study compared SS and SO in terms of mirror neurons and sensorimotor networks. However, certain limitations should be mentioned. First, participants' head motions were inevitable. For this reason, a foam pad was used to hold the head in place to minimize the head motion. Furthermore, participants with excessive head motion were discarded, and the head-motion parameters were controlled as covariates in the analysis. Before the experiment, subjects were also given a good understanding of the experiment and familiarized with the scanning environment. Second, the material we used for task A was a brushing video, while the materials for rest and task B were static symbols. The experimental materials do not match enough. Third, the rest period was also an important condition. Therefore, there are actually three conditions. In this study, we used an ABBA design. Future studies can consider Latin square design. Next, the operation of brushing cannot be accurately timed, and temporal errors are difficult to avoid. To minimize such errors, we asked the same professionally trained physiotherapist who had known in advance about the ABBA sequence to operate the brushing task. In such a way, we ensured that the brushing operator had enough psychological expectations and was prepared to brush the participants' right forearms in time. In addition, the objective evaluations are incomplete; for example, an assessment of the rubber hand effect is missing. Finally, this study was limited to healthy young adults, and future studies will need to enroll post-stroke patients to investigate the activation of sensorimotor and mirror neurons by SO or SS and to reveal motor function changes by SO or SS training.

## Conclusions

SO had a similar activation pattern as SS in healthy subjects, and it could activate more mirror neurons and brain regions related to the sensorimotor networks. Based on our preliminary study findings, SO may be a more convenient, novel, and promising alternative therapy for sensorimotor dysfunction recovery in post-stroke patients. In particular, linking the theory of mirror neurons and embodied cognition to exploring the effectiveness of sensorimotor integration rehabilitation opens a new pathway toward a comprehensive and deep understanding of neurorehabilitation mechanisms. Further large-scale clinical studies to validate the efficacy of SO therapy on functional recovery for post-stroke patients are necessary.

## Data Availability Statement

The raw data supporting the conclusions of this article will be made available by the authors, without undue reservation.

## Ethics Statement

The studies involving human participants were reviewed and approved by Ethics Committee at Yueyang Hospital of Integrated Traditional Chinese and Western Medicine, Shanghai University of Chinese Traditional Medicine, Code: 2020-178. The patients/participants provided their written informed consent to participate in this study.

## Author Contributions

ZZ, SChe, and YL participated in all studies, analyzed the data, and drafted and finalized the manuscript. CS and SCha designed the study, supervised the progression, drafted, and revised the manuscript. JZ helped to revise the manuscript critically for important intellectual content. SCha, GL, and LC helped to acquire the fMRI data. YW, SZ, and XS were involved in the recruitment of participants. XC and MR collected and sorted out the related materials. SX helped with fMRI data processing. All authors contributed to the manuscript revision and read and approved the submitted version.

## Funding

This work was supported by the National Key Research and Development Program of China (Grant No. 2018YFC2001600/04), the National Natural Science Fund (Grant No. 81874035), Shanghai Health Commission accelerated the Development of Traditional Chinese Medicine Three-Year Action Plan Project [Grant No. ZY(2018-2020)-CCCX-2001-06/2004-05], Shanghai Health Commission's Rehabilitation Diagnosis and treatment promotion project of Integrated Traditional Chinese and Western Medicine [Grant No. ZY(2018-2020)-FWTX-8002] and Intelligent Medical Program of Shanghai Health Commission (Grant No. 2018ZHYL0216).

## Conflict of Interest

The authors declare that the research was conducted in the absence of any commercial or financial relationships that could be construed as a potential conflict of interest.

## Publisher's Note

All claims expressed in this article are solely those of the authors and do not necessarily represent those of their affiliated organizations, or those of the publisher, the editors and the reviewers. Any product that may be evaluated in this article, or claim that may be made by its manufacturer, is not guaranteed or endorsed by the publisher.
