# Supplementary material for: Comparison of Sensory Observation and Somatosensory Stimulation in Mirror Neurons and the Sensorimotor Network: A Task-Based fMRI Study
Source: Front Neurol. 2022 Jun 30;13:916990. doi: 10.3389/fneur.2022.916990 (PMC9279701; doi:10.3389/fneur.2022.916990)
Supplement: Supplementary file 1 [file Data_Sheet_1.doc]

**Supplementary materials**

**TABLE S1 | A paired-sample *t* test results of task A and task B in 6 head-motion parameters.**

|  | **Mean ± SD** | ***t*-value** | ***p*-value** |
| --- | --- | --- | --- |
| **Translation (mm)** | | | |
| **X translation** | -0.0328 ± 0.0682 | -2.4520 | 0.0215**^*^** |
| **Y translation** | -0.0048 ± 0.0464 | -0.5309 | 0.6002 |
| **Z translation** | 0.0272 ± 0.0778 | 1.7834 | 0.0867 |
| **Rotation (degree)** | | | |
| **Pitch rotation** | -0.0003 ± 0.0013 | -1.2914 | 0.2084 |
| **Roll rotation** | 0.0001 ± 0.0006 | 0.8911 | 0.3814 |
| **Yaw rotation** | -0.0002 ± 0.0008 | -1.5385 | 0.1365 |

*^*^Significant difference (paired-sample t test, p* < *0.05)*

**
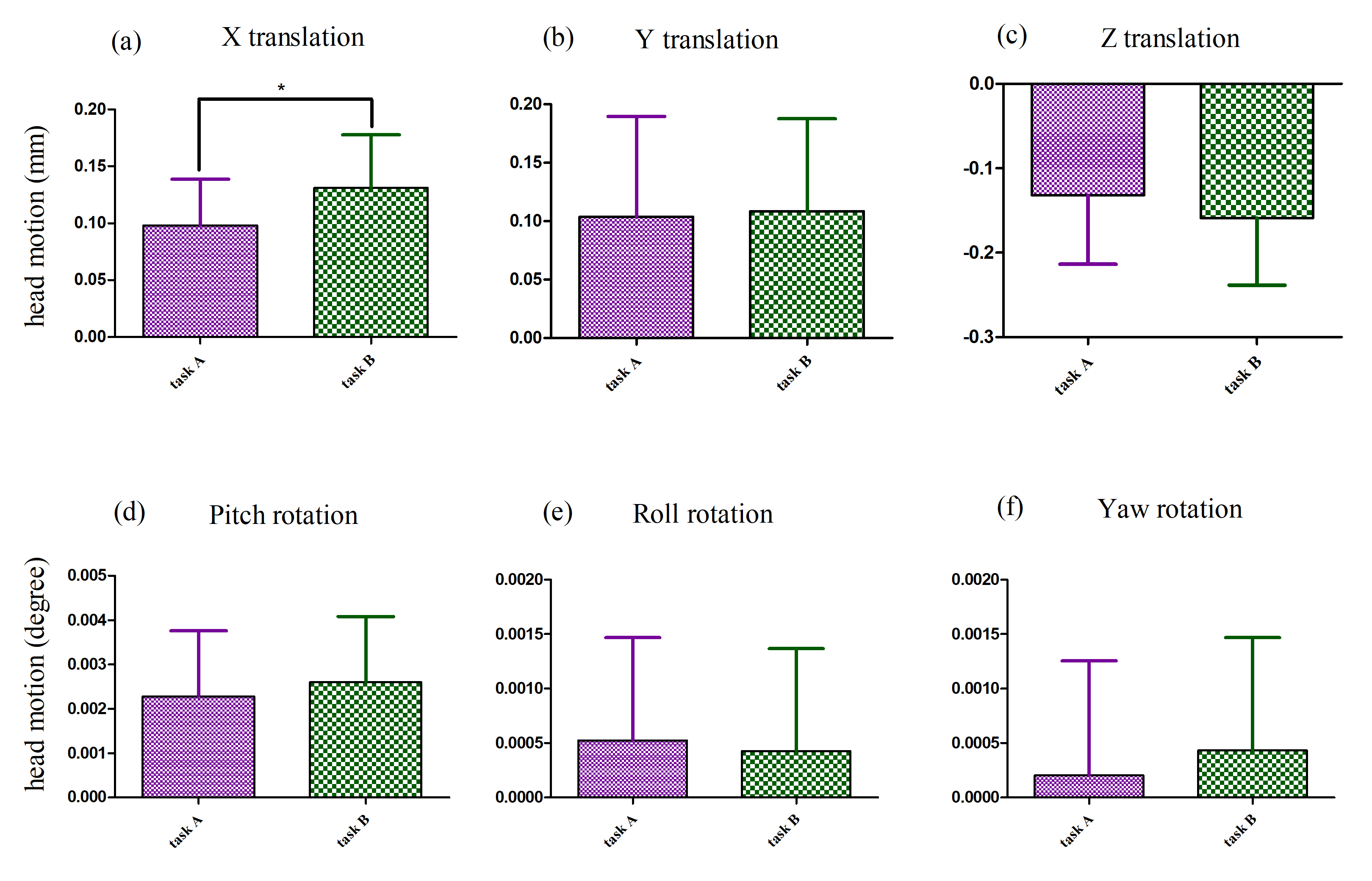
**

**FIGURE S1 |** A paired-sample *t* test results of task A and task B in 6 head-motion parameters. (a) X translation, *significant difference (paired-sample *t* test, *p* < 0.05); (b) Y translation (paired-sample *t* test, *p* *>* 0.05); (c) Z translation (paired-sample *t* test, *p* *>* 0.05); (d) Pitch rotation (paired-sample *t* test, *p* *>* 0.05); (e) Roll rotation (paired-sample *t* test, *p* *>* 0.05); (f) Yaw rotation (paired-sample *t* test, *p* *>* 0.05).
