# Supplementary figures and images for: Comparison of Sensory Observation and Somatosensory Stimulation in Mirror Neurons and the Sensorimotor Network: A Task-Based fMRI Study
Source: Front Neurol. 2022 Jun 30;13:916990. doi: 10.3389/fneur.2022.916990 (PMC9279701; doi:10.3389/fneur.2022.916990)

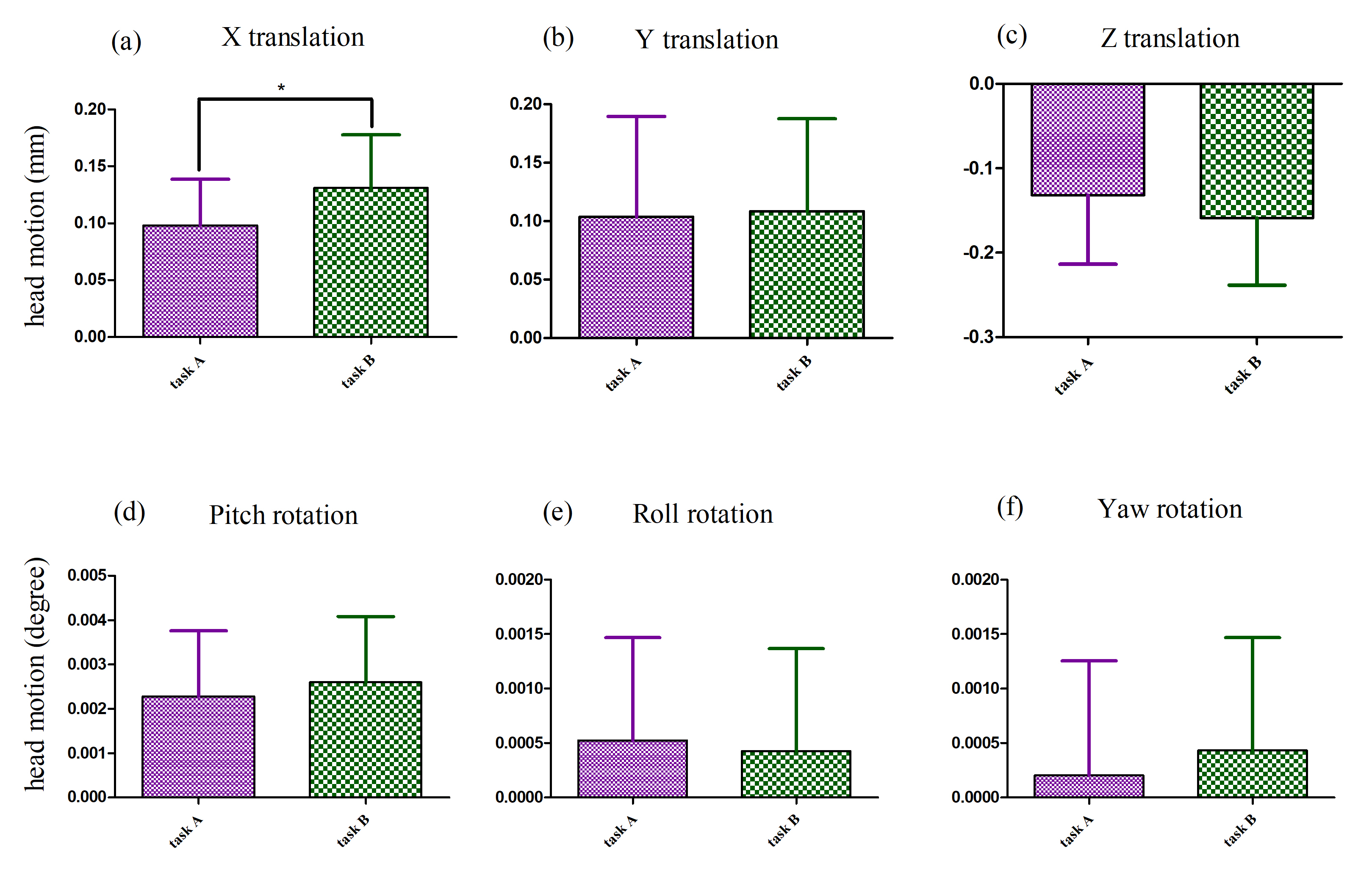

Supplement: Supplementary file 2 [file Image_1.JPEG]
